# Probing anion recognition in a cobalt(II) *de novo* designed metalloprotein^☆^

**DOI:** 10.1016/j.jinorgbio.2026.113268

**Published:** 2026-02-12

**Authors:** Salvatore La Gatta, Jacob K. Firby, James E. Penner-Hahn, Vincent L. Pecoraro

**Affiliations:** a Department of Chemistry, University of Michigan, Ann Arbor, MI 48109, USA; b Department of Biophysics, University of Michigan, Ann Arbor, MI 48109, USA

**Keywords:** *De novo* designed metalloproteins, Three-stranded coiled coil, Cobalt(II) coordination chemistry, Anion binding

## Abstract

Anions such as halides and pseudohalides influence metal-site structure and function. *De novo* designed metallocoiled coils offer a defined platform for studying how metal centers recognize small anions within an α-helical scaffold. Spectroscopic examination of anion binding to three-stranded coiled coils (3SCCs) using artificial cobalt (II) substituted carbonic anhydrases (CA) is used as an analogue of the zinc(II) center. Scaffolds composed of three equivalents of GRW-H (Ac-GWKALEEKLKALEEKLKALEEKLKALEEKHKALEEKG-NH_2_) yield a cobalt(II) (His)_3_ site whose visible spectrum can be perturbed by nitrite, azide, and thiocyanate, producing significant ligand-field spectral changes that reveal these ions bind with millimolar affinities. These modifications reflect similar chemistry to that observed for cobalt(II)-substituted *CA*. X-ray absorption spectroscopy confirms that thiocyanate coordinates through nitrogen, converting a 6-coordinate cobalt(II)(His)_3_(H_2_O)_3−x_(OH^−^)_x_ (with x = 0 or 1) species at pH ≤ 9 to a five-coordinate cobalt(II) center. pH-dependent measurements reveal a factor of 2 affinity increase for thiocyanate binding as solution basicity increases, with a pKa ~ 8.0 consistent with a single deprotonation event. This strengthening of the binding constant does not arise from thiocyanic acid acidity or cobalt hydrolysis and likely reflects deprotonation of a protein residue(s). In contrast to thiocyanate or azide, halides (chloride through iodide) bind much more weakly. The spectral parameters observed vary with anion properties and reflect distinct cobalt(II) geometries. Overall, these results define how a simple His_3_ site embedded in a designed protein scaffold recognizes anions and adopts distinct geometries, providing a foundation for designing metalloproteins that activate small inorganic substrates.

## 1. Introduction

Harry Gray has been a monumental figure in the field of Inorganic chemistry, in particular for his contributions to defining spectroscopic and electronic structures of transition ion complexes to understand metal dependent processes in biology. His interests have ranged from studies of biological long range electron transfer [1,2] to clarifying the nature of bonding modes of ligands to metal ions such as his oxo wall proposal for the reactivity of M = O enzymatic catalysts [3,4]. While often focusing on Fe [5,6] and Cu [7,8] systems, his work has traversed the periodic table. Relevant to the chemistry presented herein, he provided insight for the binding and influence of anions to the spectroscopic and magnetic properties of anions to Co(II) [9] and explored the chemistry of hydrolytic enzymes such as carboxypeptidase using Co(II) substitution [10].

Anions (such as halides, nitrites and pseudohalides) often guide the chemistry of metalloproteins. They may tune reactivity, increase the stability, or serve as substrates in catalysis when bound to metal centers. Their size, polarizability and the cost of removing their hydration shell influence their recognition by the metal center. Studying these principles helps us understand natural systems, while also empowering us to design artificial metalloproteins.

To go beyond the limits of structure and functions set by natural evolution, researchers can design metalloproteins from scratch by using a *de novo* design approach [11-15]. This method allows one to dissect and study the individual interactions that influence the active sites of natural metalloproteins and systematically investigate how precise interactions can modify the functions we see in these systems. This approach has enabled the design of systems that replicate the structural and functional features of natural metalloenzymes, including mononuclear [16-21], dinuclear [22-27] and even multinuclear metal sites [28-30] displaying a diverse range of catalytic activities [31-37]. In our laboratory, we have mainly focused on coiled coils [38,39] and helical bundle scaffolds [40,41], which provide geometrically defined environments suitable for engineering metal-binding sites [42].

Through these scaffolds, we have engineered copper-dependent activities, most notably superoxide dismutation [43] and nitrite reduction [44] and zinc and cobalt enzymes capable of CO_2_ hydration [45,46] and ester hydrolysis [17]. Most of these reactions rely on small anionic substrates. Studying their interaction with the metal center directly, however, can be difficult. Superoxide, for example, is extremely short-lived and not easily handled in solution. In order to get around this problem, chemists often use stable mimics like azide, which has the same electronic structure and shape. Nitrite, on the other hand, is much less reactive and can be directly tested under most experimental conditions. Similarly, bicarbonate is very difficult to directly monitor through its dehydration reaction.

To tune the affinity and catalytic efficiency toward these substrates, we introduced systematic modifications around the metal-binding site of the 3-stranded coiled coil (3SCC) and 3-helix bundle (3HB) scaffolds, leading to a deeper understanding of the impact of the protein environment on the displayed reactivity. Introducing electrostatic effects by putting charged amino acids next to the coordination site and tweaking the space around the catalytic pocket with Leu to Ala substitutions, allowed us to uncover marked changes in metal-binding affinity, spectroscopic features, redox potentials, and most importantly affinity and catalytic activity toward anionic substrates. Building on this foundation, we sought to address the complementary question of how different anions influence the coordination geometry, electronic structure, and binding properties of the holo-3SCC complex.

**Fig. 1.**
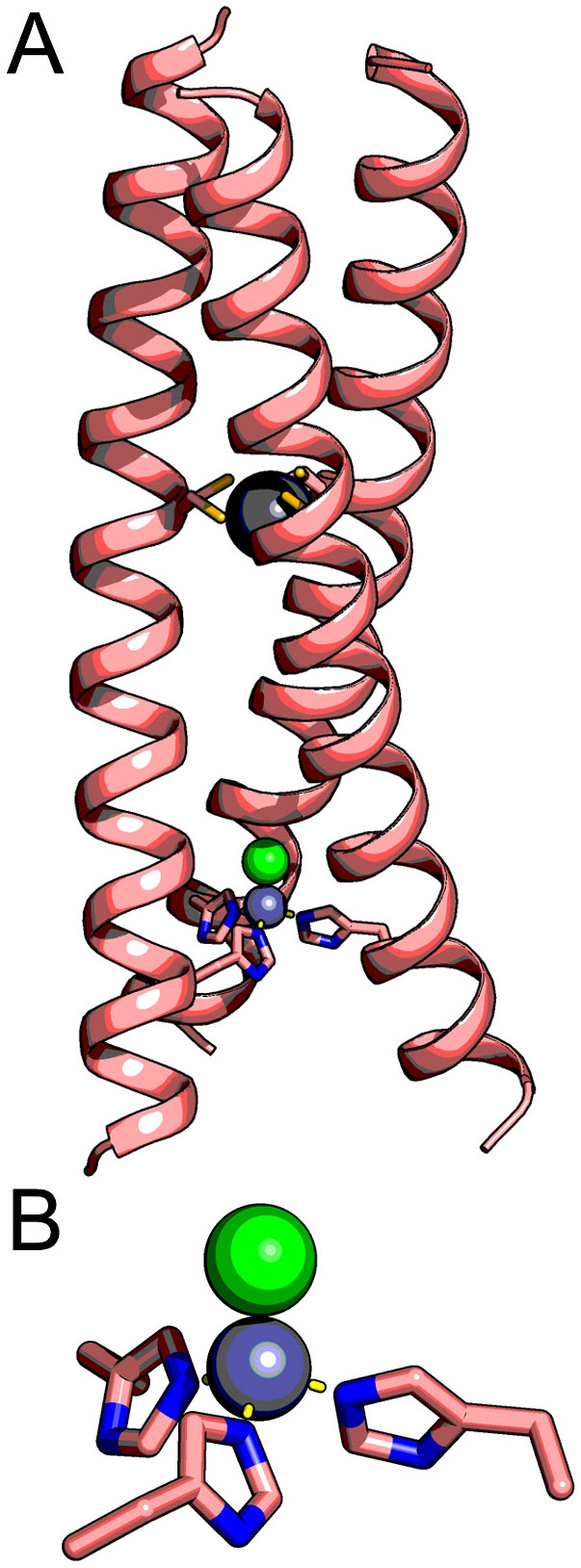
Structural representation of the 3SCC scaffold and its transition metal-binding site based on the X-ray crystal structure of Pb(II)Zn(II)-GRCS-L16C-L30H (PDB ID: 5kb0). (A) Ribbon representation of the 3SCC showing the overall trimeric architecture. (B) Close-up view of the transition metal-binding site, illustrating the Zn(II) center (gray sphere) coordinated by three histidine residues (shown as sticks) and one chloride ligand (green sphere).

Herein, we explore anion binding to *de novo* designed mimics for carbonic anhydrase (CA) activities. Previously, we reported X-ray structures of highly active 3SCC containing Zn(II) at a tris histidine binding site. The transition ion is bound to three histidines, one from each α-helical strand, and contains one exogenous ligand, either water or chloride (Fig. 1). Unfortunately, this d^10^ ion cannot be interrogated by numerous spectroscopic techniques; however, Co(II) substitution often allows examination of the active site using an ion that retains catalytic activity. Due to its d^7^ electronic configuration, Co(II) serves as a spectroscopic probe offering high sensitivity to ligand-field perturbations and a convenient way to follow anion binding at the metal site.

In this study, we investigate the binding of small anions to a Co(II)-3SCC complex, here referred to as Co(II)-(GRW-H)_3_, as a spectroscopic analogue of our Zn(II)-3SCC complex that displays catalytic hydrolytic activity with rates approaching those of natural CA [17,45]. Our motivation finds foundation from the chemistry of zinc-dependent CAs, where anions such as halides, pseudohalides (*e.g.*, $N_{3}^{-}$, NCS^−^), and oxyanions like nitrite (${\text{NO}}_{2}^{-}$) have crucial roles in tuning the equilibrium between hydration and dehydration reactions [47]. In this context, Co(II) substitution allows direct spectroscopic interrogation of the active site, offering insight into ligand-field perturbations that guide anion binding [48-51]. Nitrite acts as a substrate analog for Zn(II)- and Co(II)-based hydrolytic enzymes [52], although its role in nitrite-dependent NO formation and vasodilation remains a subject of debate [53]. Azide and thiocyanate allow for the evaluation of donor atom preferences and the geometric adaptability of the Co(II) metal site. Furthermore, to assess the effects of ionic radius and polarizability on binding affinity and coordination geometry preferences, halide binding was evaluated. Overall, these results provide a framework for understanding how a *de novo* designed three-histidine metal binding site within a 3SCC scaffold interacts with anions, shedding light on the characteristics that underlie hydrolytic reactivity in both natural and artificial systems.

## 2. Materials and methods

### 2.1. Materials

All Fmoc (9-fluorenylmethoxycarbonyl) protected amino acids and Rink amide 4-methylbenzhydrylamine (MBHA) were purchased from Aapptec. Coupling reagents (N,N-diisopropylcarbodiimide (DIC) and ethyl cyanohydroxyiminoacetate (Oxyma)) were purchased from Sigma Aldrich. Acetic anhydrase was purchased from Thermo Fisher Chemicals. All solvents and scavengers used in the synthesis and purification (*N*,*N*-Dimethylformamide (DMF), piperidine, TFA, acetonitrile, triisopropysilane, diethyl ether, dichloromethane) were supplied by Thermo Fisher Chemicals and/or Sigma Aldrich. Cobalt(II) sulfate heptahydrate, 4-(2-Hydroxyethyl)piperazine-1-ethanesulfonic acid (HEPES), and N-cyclohexyl-2-aminoethanesulfonic acid (CHES) were purchased from Sigma Aldrich. Waters supplied the XBridge Peptide BEH C18 OBD preparative column. UV–Vis electronic absorption spectroscopy analyses were performed with OriginPro 9.6.5 software.

### 2.2. Peptide synthesis

GRW-H peptide was synthesized by automatic solid-phase synthesis (Liberty Blue 2.0 peptide synthesizer – CEM corporation) on a 0.1 mmol scale. An Fmoc-Rink Amide MBHA resin with a substitution level of 0.49 mmol/g was used as the solid support for synthesis and was swelled with dichloromethane before the synthesis. Deprotection and coupling steps were repeated with each subsequent amino acid until the peptide chain assembly was completed. Coupling steps were achieved with the coupling reagents DIC in DMF (1 M) and oxyma in DMF (1 M) at 90 °C for 2 min. Deprotection steps were performed using 10% (v/v) piperidine in DMF at 90 °C for 1 min. To prevent epimerization during the coupling step, Fmoc-His(Trt)-OH was coupled at 50 °C for 10 min. The N-terminus amino group was acetylated with 10% (v/v) acetic anhydride in DMF. After the synthesis, the resin was washed with dichloromethane and diethyl ether and finally dried. The peptide cleavage from the resin, with concomitant side chain deprotection, was carried out with a solution (30 mL) of a mixture of 94:3:3 (v/v) TFA, triisopropylsilane, and water for 2 h at room temperature, with slight magnetic stirring. The resin was filtered, and the solution was collected under vacuum in a glass flask, then the resin was washed again 2 times with only TFA. The volume of the solution was then reduced to about 5 mL or lower using a N_2_ flow. The cleaved peptide was precipitated by adding 40 mL of cold diethyl ether and recovered by vacuum filtration. The precipitate was washed three times with 20 mL of cold diethyl ether. The precipitated peptide was dried under vacuum, re-dissolved in water, and lyophilized, to yield an off-white powder. GRW-H peptide was obtained in about 90% yield, based on the resin substitution level. Peptide identity was assessed by high resolution mass spectrometry analysis, using an Agilent 6230 time-of-flight (TOF) mass spectrometer, using electrospray ionization (ESI) as ion source, operated in positive mode. The lyophilized crude peptide was then redissolved in water with 0.1% (v/v) TFA and loaded onto a chromatographic column XBridge Peptide BEH C18 OBD preparative column (pore size 300 Å, particle size 10 μm, diameter 19 mm, length 250 mm) using a Waters 600E HPLC system connected to a Waters 486 tunable absorbance detector. The eluents were H_2_O with 0.1% (v/v) TFA (eluent A) and acetonitrile:H_2_O 90:10 with 0.1% (v/v) TFA (eluent B) and the flow rate was 20 mL min^−1^. The elution was performed with a linear gradient of eluent B from 5% to 80% over 50 min, appropriately scaled up. The peptide identity and purity in the collected fractions were verified by mass spectrometry. The pooled fractions, containing the desired peptide, were then lyophilized and stored at −80 °C until use.

### 2.3. UV–Vis spectroscopy

UV–Vis absorption spectra were recorded with a Cary Varian 5000 Spectrophotometer, equipped with a thermostatic cell compartment (Varian, Palo Alto, CA, USA), using a quartz cuvette with 1 cm path length. Wavelength scans were performed at 25 °C from 350 to 800 nm, with a 600 nm min^−1^ scan speed. GRW-H peptide was dissolved in Milli-Q water at a concentration of about 60 mg/mL (about 10 mM monomer). The stock solution concentration of GRW-H peptide was determined spectrophotometrically monitoring the tryptophan residue by using ε_280nm_ = 5500 M^−1^ cm^−1^ [54].

Stock solutions of anions were prepared from the corresponding salts at the following concentrations: NCS^−^ from sodium thiocyanate (NaSCN, 10 M), $N_{3}^{-}$ from sodium azide (NaN_3_, 5 M), ${\text{NO}}_{2}^{-}$ from sodium nitrite (NaNO_2_, 3.27 M), F^−^ from potassium fluoride (KF, 10 M), Cl^−^ from sodium chloride (NaCl, 5 M), Br^−^ from sodium bromide (NaBr, 7 M), and I^−^ from potassium iodide (KI, 10 M). All anion salts were dried at room temperature in a desiccator until no further change in weight was observed. Stock solution of iodide was verified to contain no detectable triiodide ($I_{3}^{-}$), as confirmed by the absence of the characteristic ~ 350 nm band associated with $I_{3}^{-}$ formation.

For NaSCN, NaN_3_, and NaNO_2_ titrations, solutions of Co(II)-(GRW-H)_3_ were prepared to yield final concentrations of 0.3 mM (GRW-H)_3_ and 0.24 mM CoSO_4_ in 200 mM CHES buffer at pH 9.0, as follows. The concentrated GRW-H peptide stock solution was diluted in 200 mM CHES buffer at pH 9.0. During this step, the pH was decreased to approximately 8.5. CoSO_4_ was then added from a 200 mM aqueous stock solution previously quantified by ICP-MS. The pH was carefully readjusted to 9.0 with small volumes of concentrated NaOH or H_2_SO_4_. NaOH solutions were freshly prepared before use, as aged solutions can absorb atmospheric CO_2_ and form sodium carbonate, which may interfere with UV–Vis spectroscopic measurements. Incremental additions of anion stock solutions were performed until the final anion concentrations were reached (1030 mM for NaSCN, 730 mM for NaN_3_, and 775 mM for NaNO_2_). After each addition, the solution was gently mixed for 10 min and its UV–Vis spectrum was recorded. At the end of each titration, the pH of the solution was checked and found to remain unchanged. Each spectrum was corrected by subtracting the apo-(GRW-H)_3_ spectrum at the same concentration (0.3 mM) as in the Co(II)-(GRW-H)_3_ solution, and all spectra were further corrected for dilution effects. Because halide binding required higher anion concentrations to reach saturation, the titrations for Cl^−^, Br^−^, and I^−^ were performed using a modified procedure. Two solutions were prepared: one containing 0.24 mM CoSO_4_, 0.3 mM (GRW-H)_3_ and 200 mM CHES buffer at pH 9.0 and another at the highest concentration of each halide (3300 mM for Cl^−^, 4620 mM for Br^−^, 2660 mM for I^−^) and 0.24 mM CoSO_4_, 0.3 mM (GRW-H)_3_ and 200 mM CHES buffer at pH 9.0. The pH values of both solutions were carefully adjusted with small volumes of concentrated NaOH or H_2_SO_4_. The UV–Vis spectrum of the anion-free solution (containing only Co(II)-(GRW-H)_3_) was first recorded. Subsequently, small aliquots of this solution were replaced with equal volumes of the concentrated anion-containing solution. After each addition, the mixture was gently mixed and then allowed to equilibrate for 10 min before recording the spectrum. Each spectrum was corrected by subtracting that of the apo-(GRW-H)_3_ at the same concentration (0.3 mM) as in the Co(II)-(GRW-H)_3_ solution.

The equilibrium between the $\text{Co(II)-}{\left(\text{GRW-}H\right)}_{3}-L$ and $\text{Co(II)-}{\left(\text{GRW-}H\right)}_{3}+L$ (where L is the anion) can be represented as:

$$\text{Co(II)-}{\left(\text{GRW-}H\right)}_{3}-L⇄\text{Co(II)-}{\left(\text{GRW-}H\right)}_{3}+L.$$

Binding isotherms for this equilibrium were obtained by plotting the change in absorbance (ΔA), defined as the difference between the absorbance of the Co(II)-(GRW-H)_3_–anion complex at the corresponding $\lambda_{\max}$ (605 nm for NCS^−^, 588 nm for $N_{3}^{-}$, 562 nm for ${\text{NO}}_{2}^{-}$, 587 nm for Cl^−^, 594 nm for Br^−^, and 603 nm for I^−^) and that of Co(II)-(GRW-H)_3_ in the absence of anion, as a function of anion concentration. The experimental data were fitted using a one-site binding model according to the following equation:

$$y=\frac{A_{1}x+A_{0}K_{D}}{K_{D}+x}$$

where y is the absorbance at a given anion concentration x, $A_{0}$ is the absorbance in the absence of anion, $A_{1}$ is the absorbance at saturation, and $K_{D}$ is the apparent dissociation constant derived from the above equilibrium. Non-linear least-squares fitting was performed using OriginPro 9.6.5 software. Deconvolution of the visible spectra of Co(II)-(GRW-H)_3_-anion complexes was performed using the Peak Analyzer function in OriginPro 9.6.5 software to separate overlapping d-d transition bands. Baseline correction and Gaussian fitting were applied to determine the position and intensity of each component peak.

The $K_{D}$ values for NCS^−^ were determined at different pH values (7.0, 7.5, 8.0, 8.6, and 9.0) under identical experimental conditions (using 200 mM HEPES as buffer for pH 7, 7.5, 8.0 and 200 mM CHES as buffer for pH 8.6 and 9). The $K_{D}$ values were plotted as a function of pH, and the data were fitted using an acid–base equilibrium according to the following equation:

$$y=K_{D,\text{min}}+\frac{\left(K_{D,\max}-K_{D,\text{min}}\right)}{1+10^{n({\text{pK}}_{a}-x)}}$$

where y is the apparent dissociation constant at a given pH x, $K_{D,\text{min}}$ and $K_{D,\max}$ are the limiting dissociation constants at low and high pH, respectively, n corresponds to the number of protons involved in the equilibrium, and pK_a_ represents the apparent ionization constant of the group involved in the binding equilibrium. Non-linear least-squares fitting was performed using OriginPro 9.6.5 software.

Structural models were generated starting from the X-ray crystal structure of Pb(II)Zn(II)-GRCS-L16C-L30H (PDB ID: 5kb0), used as an analog of GRW-H. The atomic coordinates of the parent structure were maintained throughout the model construction. For tetrahedral coordination models, the Cl^−^ ligand, present in the crystal structure as a fourth ligand of the metal, was replaced with I^−^ or NCS^−^, using metal-ligand bond distances reported in the literature (2.5 Å for I^−^ and 2.1 Å for NCS^−^). For pentacoordinate models, I^−^ or NCS^−^ and an additional water molecule were introduced. The anions and the water molecules were manually positioned to generate sterically reasonable coordination geometries within the binding cavity, so minimizing unfavorable contacts with surrounding Leu26 residues. These models are intended solely to provide qualitative visualizations of plausible coordination arrangements and do not represent experimentally determined or energetically optimized structures.

### 2.4. XAS spectroscopy

A sample containing final concentrations of 1 mM CoSO_4_, 1.5 mM (GRW-H)_3_, 50 mM CHES buffer, 30% (v/v) glycerol, and 500 mM NaSCN at pH 9.0 was prepared as follows. A concentrated GRW-H peptide stock solution (in water) was diluted in 50 mM CHES buffer at pH 9.0. During dilution, the pH of the solution dropped to approximately 5.0 and was carefully readjusted to around 7.5 by small additions of concentrated NaOH or H_2_SO_4_. CoSO_4_ was then added from a 200 mM aqueous stock solution previously quantified by ICP-MS. The mixture was allowed to equilibrate for about 15 min, after which the pH was adjusted to 9.0. A small aliquot of 10 M NaSCN solution was added, turning the solution color from pale pink to a faint bluish shade, indicative of the formation of the Co(II)-(GRW-H)_3_-NCS^−^ complex. The sample was then allowed to equilibrate, after which glycerol was added to reach a final concentration of 30% (v/v), and the mixture was left to equilibrate under gentle stirring for 15 min. Finally, the sample was loaded into a sample cell and flash-frozen in liquid nitrogen. Measurements were carried out at Stanford Synchrotron Radiation Lightsource (SSRL) beamline 7–3 with an Si (220) double-crystal monochromator and a flat Rh-coated harmonic rejection mirror. Samples were maintained below 10 K with an Oxford Instruments liquid helium cryostat. Data were measured as fluorescence excitation spectra using a 30-element Ge array detector equipped with an iron low-pass filter and Soller slits, normalized to incident intensity measured with an N_2_-filled ion chamber. Data were measured with steps of 0.35 eV in the XANES region (1 s integration time) and 0.05 Å^−1^ in the EXAFS region to *k* = 13 Å^−1^ (1.5–28 s integration, *k*^3^–weighted). Energies were calibrated by assigning the lowest energy inflection point of a cobalt metal foil as 7708.9 eV. XANES data were normalized with Athena [55] and processed and the background was removed using a 3-region cubic spline. The EXAFS data were analyzed using Artemis [55].

## 3. Results

To establish a well-defined Co(II) coordination site, we employed a *de novo* designed 3SCC, (GRW-H)_3_, engineered to present one histidine residue from each helix toward the interior of the bundle. The sequence follows the canonical heptad repeat (*a b c d e f g*), where hydrophobic residues at the *a* and *d* positions drive trimer formation through knobs-into-holes packing [56]. Electrostatic interactions between residues at the *e* and *g* positions stabilize the parallel orientation of the helices, while residues at the *b*, *c*, and *f* positions contribute to overall stability and modulate peptide solubility. The peptide consists of five heptads based on the L K A L E E K heptad, flanked by an N-terminal and a C-terminal glycine. A His residue was introduced at the *a* position of the fifth heptad to generate the metal-binding site, and a Trp residue was placed at the *a* position of the first heptad for spectroscopic quantification.

The peptide was synthesized using standard Fmoc-based solid-phase peptide synthesis (SPPS), acetylated at the N terminus and amidated at the C terminus, purified by reverse-phase HPLC, and its identity was confirmed by electrospray ionization mass spectrometry (Fig. S1).

The final sequence is Ac-GWKALEEKLKALEEKLKALEEKLKA-LEEKHKALEEKG-NH_2_.

To investigate how different anions modulate the coordination environment of Co(II)-(GRW-H)_3_, we combined UV–Vis absorption spectroscopy with X-ray absorption spectroscopy (XAS) and binding affinity analysis. All measurements were performed at pH 9.0, unless stated otherwise.

### 3.1. UV-Vis spectroscopy

**Fig. 2.**
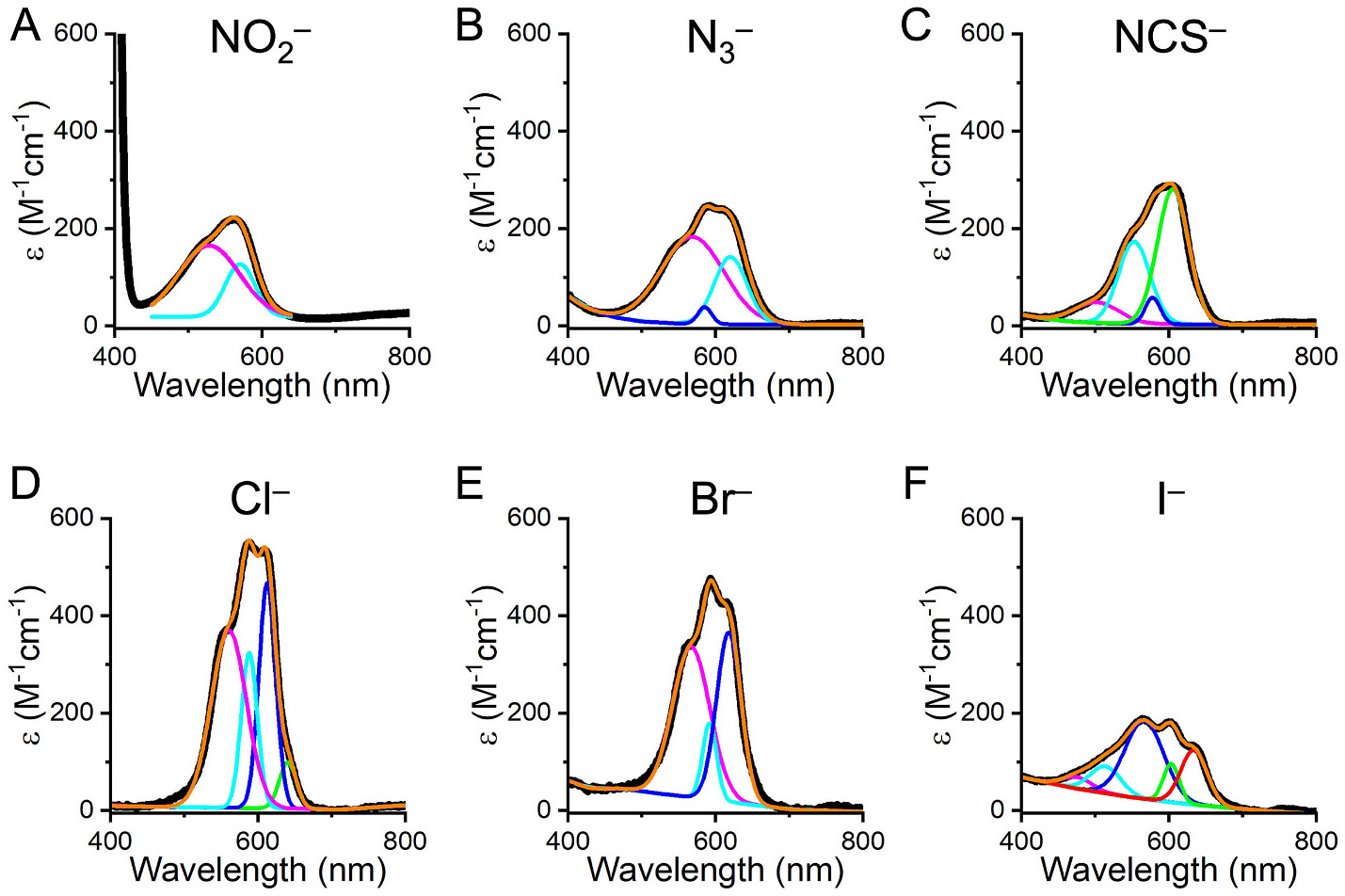
Visible electronic absorption spectra of Co(II)-(GRW-H)_3_ (0.24 mM) in the presence of various anions. (A) Co(II)-(GRW-H)_3_ + 775 mM NaNO_2_; (B) Co(II)-(GRW-H)_3_ + 525 mM NaN_3_; (C) Co(II)-(GRW-H)_3_ + 575 mM NaSCN; (D) Co(II)-(GRW-H)_3_ + 3300 mM NaCl; (E) Co(II)-(GRW-H)_3_ + 4620 mM NaBr; (F) Co(II)-(GRW-H)_3_ + 2660 mM KI. Black traces represent the experimental spectra collected; colored lines correspond to the individual components from spectral deconvolution, and the orange trace represents the cumulative fit derived from their sum. All spectra were recorded in 200 mM CHES, pH 9.0.

**Fig. 3.**
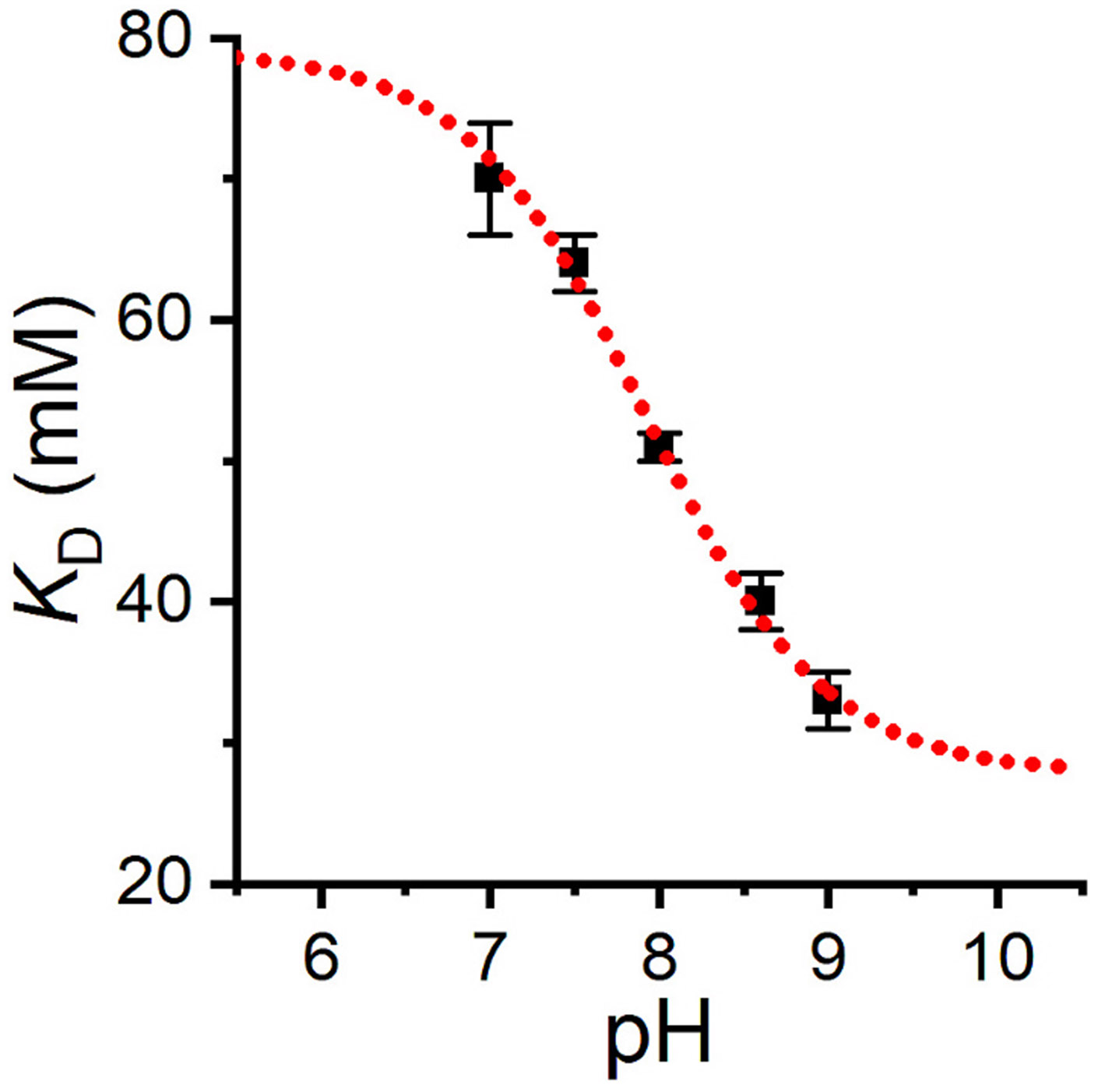
pH dependence of the apparent dissociation constant (*K*_D_) for NCS^−^ binding to Co(II)-(GRW-H)_3_. Experimental *K*_D_ values obtained at different pH values are plotted as black square, while the red dotted curve represents the best fit to an acid-base equilibrium model considering the uptake or release of n protons during binding.

Upon binding of anionic ligands, significant changes were observed in the cobalt d-d electronic transition region, allowing characterization of ligand-induced perturbations and quantification of apparent dissociation constants ($K_{D}$) using a single-site binding model (Fig. S2). In the absence of exogenous ligands, Co(II)-(GRW-H)_3_ exhibited a single broad absorption band centered at 511 nm with low molar absorptivity (ε ≈ 40 M^−1^ cm^−1^) (Fig. S3), consistent with a six-coordinate environment involving three histidine residues and three water molecules. Addition of sodium nitrite (NaNO_2_) produced a distinct absorption maximum at 562 nm with significantly increased intensity (ε = 220 M^−1^ cm^−1^), consistent with nitrite coordination to the Co(II) center and a change in ligand field geometry. The apparent dissociation constant was estimated at (108 ± 2) mM. Deconvolutions identify 2 main peaks at 528 nm (ε = 165 M^−1^ cm^−1^) and 570 (125 M^−1^ cm^−1^) (Fig. 2A and Table 1). Addition of sodium azide (NaN_3_) produced clear spectral changes. Deconvolution identified three transitions at 569 nm (ε = 180 M^−1^ cm^−1^), 586 nm (ε = 40 M^−1^ cm^−1^), and 620 nm (ε = 140 M^−1^ cm^−1^) (Fig. 2B and Table 1), with an apparent *K*_D_ estimated at (34 ± 2) mM. Addition of sodium thiocyanate (NaSCN) also induced marked spectral structuring. Deconvolution revealed four d-d transitions centered at 500 nm (ε = 40 M^−1^ cm^−1^), 552 nm (ε = 168 M^−1^ cm^−1^), 578 nm (ε = 55 M^−1^ cm^−1^), and 605 nm (ε = 280 M^−1^ cm^−1^) (Fig. 2C and Table 1). The apparent *K*_D_ for NCS^−^ binding was determined to be (33 ± 2) mM. Nitrite, NCS^−^, and $N_{3}^{-}$ all induced pronounced ligand-field perturbations, yielding more structured absorption features compared to Co(II)-(GRW-H)_3_. In addition, pH-dependent binding studies were performed for NCS^−^. The apparent *K*_D_ decreased with increasing pH, and fitting to a one-proton model yielded a pK_a_ of (8.0 ± 0.09) (Fig. 3). The analysis then shifted from the more polarizable pseudohalides to the less polarizable halides, in order to evaluate how decreasing polarizability and increasing charge localization influence anion binding within the Co(II)-(GRW-H)_3_ scaffold. An additional objective of examining halides was to assess the affinity of small monoatomic anions lacking oxygen or nitrogen donor atoms, thereby isolating the effect of charge and size on metal-anion interactions. Upon fluoride addition, no spectroscopic changes are observed, suggesting no interaction with the metal center (Fig. S4). On the other hand, chloride binding displays broader transitions but with higher molar absorptivities relative to the pseudohalide-bound complexes. Deconvolution resolved four bands at 560 nm (ε = 365 M^−1^ cm^−1^), 588 nm (ε = 320 M^−1^ cm^−1^), 613 nm (ε = 460 M^−1^ cm^−1^), and 640 nm (ε = 95 M^−1^ cm^−1^) (Fig. 2D and Table 1). The apparent *K*_D_ for Cl^−^ binding was (545 ± 21) mM. Bromide addition gave more diffuse spectral features, with broadening and partial overlap of transitions. Three bands were resolved at 567 nm (ε = 335 M^−1^ cm^−1^), 592 nm (ε = 180 M^−1^ cm^−1^), and 618 nm (ε =360 M^−1^ cm^−1^) (Fig. 2E and Table 1). The apparent *K*_D_ was estimated at (1108 ± 38) mM. At high concentrations of sodium iodide (4620 mM), the spectrum displayed five relatively well-resolved transitions at 471 nm (ε = 70 M^−1^ cm^−1^), 514 nm (ε = 90 M^−1^ cm^−1^), 567 nm (ε = 185 M^−1^ cm^−1^), 603 nm (ε = 95 M^−1^ cm^−1^), and 634 nm (ε = 125 M^−1^ cm^−1^) (Fig. 2F and Table 1). The apparent *K*_D_ was determined to be (1046 ± 36) mM. It should be noted that for the halides, full saturation was not reached within the experimentally accessible concentration range; therefore, the reported *K*_D_ values should be regarded as lower-limit estimates of the true dissociation constants.

### 3.2. XAS spectroscopy

**Fig. 4.**
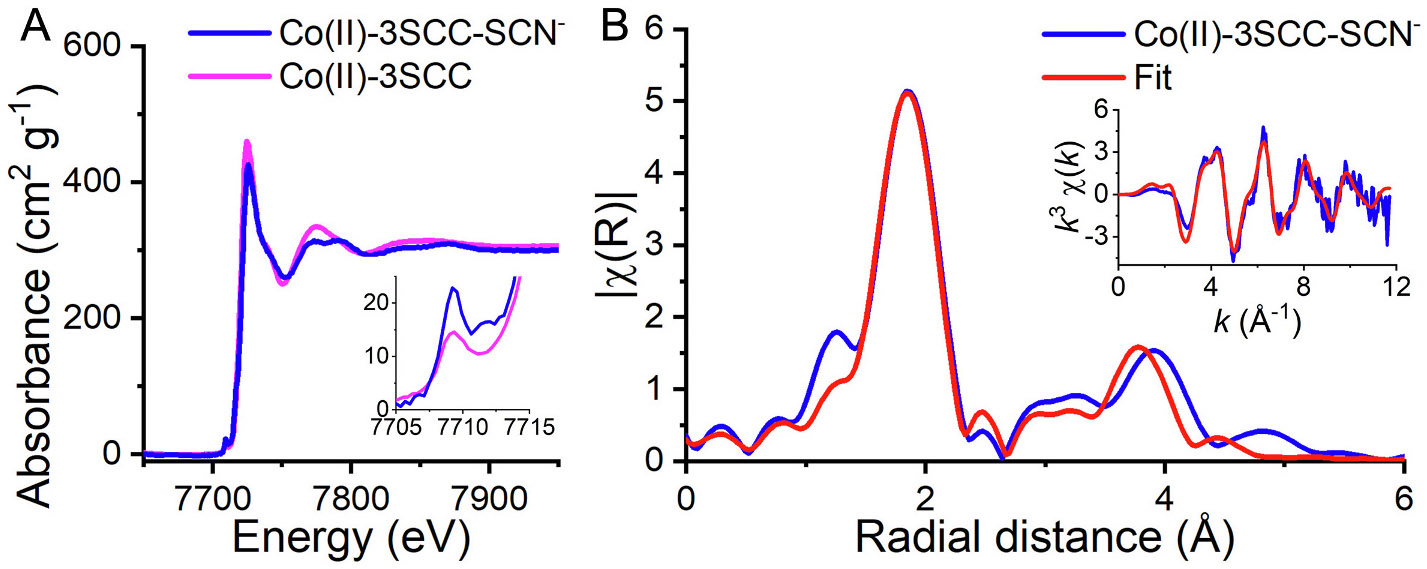
XAS of Co(II)-(GRW-H)_3_-NCS^−^ and a reference construct. (A) Normalized XANES spectra: Co(II)-(GRW-H)_3_-NCS^−^ (pH 9.0, blue) and Co(II)-(TRIW-H)_3_ (reference) (pH 9.5, magenta); inset: pre-edge region. (B) Phase-uncorrected Fourier transform of the EXAFS region for the Co(II)-(GRW-H)_3_-NCS^−^ (blue) with best-fit model (red) that includes outer-shell scattering contributions from both thiocyanate and the three coordinating histidine residues; inset: *k*^3^-weighted EXAFS data (blue) with best-fit model (red) in *k*-space. Co(II)-(GRW-H)_3_ and Co(II)-(TRIW-H)_3_ display similar spectroscopic behavior in solution; the only difference between the 3SCCs is the peptide length.

We employed Co K-edge XAS to probe the first coordination environment of the Co(II)-(GRW-H)_3_-NCS^−^ complex. Thiocyanate is an ambidentate ligand that can coordinate through either nitrogen or sulfur. Since Co(II) is a borderline hard/soft Lewis acid, it is capable of binding thiocyanate through either donor atom. XAS measurements provide a means to distinguish these possibilities owing to the stronger scattering of sulfur compared to nitrogen. A notable change in the pre-edge feature is revealed by the X-ray Absorption Near Edge Structure (XANES) region of the spectrum. The intensity of the 1 s ➝ 3d pre-edge peak is significantly larger in the NCS^−^-bound complex than in Co(II)-(GRW-H)_3_ (Fig. 4A). This increase is consistent with a shift from a more centrosymmetric octahedral environment to a less symmetric coordination geometry upon thiocyanate binding. Extended X-ray absorption fine structure (EXAFS) data are most consistent with NCS^−^ binding through the nitrogen in Co(II)-(GRW-H)_3_-NCS^−^. The data are well modeled using a single shell of 5 O/N ligands at 2.07 Å (Table S1). While it is possible to fit the data with models that include Co–S scattering, the improvement in fit quality is modest (~3-fold) for a doubling of the number of variable parameters. More importantly, mixed N + S fits gave chemically nonsensical fit parameters (Table S1): apparent Co–N coordination number of 2, unrealistically small Co–N Debye-Waller factor (9 × 10^−4^ Å^2^), unrealistically large Co–S Debye-Waller factor (1.1 × 10^− 2^ Å^2^), and unrealistically short Co–S bond-length (1.75 Å). A final fit, including the outer shell scattering from both the thiocyanate and three histidines is shown in Fig. 4B, and the corresponding fitting parameters for the outer shell scattering are reported in Table S2.

## 4. Discussion

The field of *de novo* designed metalloproteins has developed through addressing key issues in biophysics and bioinorganic chemistry [57-60]. Early studies focused on developing robust scaffolds composed of desired secondary structural elements such as α-helices using primary sequences that were unknown in natural proteins [61]. Once such structures were established, metal binding sites were engineered into the resulting scaffolds with the intent to control the number, types and orientations of metal coordination ligands provided by amino acid sidechains [62-64]. Subsequently, significant effort has been expended generating systems that mimic the structure or function of natural proteins, whether that be for metal recognition [65-68], electron transfer reactions [69-73] or catalysis [74-79]. More recently, these latter systems are being optimized by altering the metal outer sphere environment [80-85]. However, another important property defining the rate and selectivity of biological metal catalysis is the influence of anions that may serve either as a co-catalyst or as an inhibitor of the desired chemical reaction. Detailed studies exploring the interaction of common anions with *de novo* designed metalloproteins have not been extensively explored.

In contrast, anion binding has been shown to be one of the key features of the way metalloproteins operate and has been studied quite deeply. Anions are often the desired substrate in chemical catalysis (*e.g.*, ${\text{NO}}_{3}^{-}$, ${\text{NO}}_{2}^{-}$, ${\text{SO}}_{3}^{-}$, ${\text{HCO}}_{3}^{-}$ and $O_{2}^{-}$). Even when not directly transformed by the metalloenzyme, anions may help control substrate recognition, speed up the catalytic process or contribute to structural stability.

Directly probing these interactions, however, is often challenging because some biologically relevant metal centers do not lend themselves easily to spectroscopic analysis. For example, Zn(II), as a consequence of its d^10^ electronic configuration, is silent in numerous spectroscopic techniques. To skirt this practical limitation Co(II) has proven to be a valuable surrogate for Zn(II) as it often can retain significant catalytic activity when substituted into a Zn protein and may even adopt the same or similar coordination structure [86]. Because Co(II) has a d^7^ electronic configuration, electronic transitions that are highly sensitive to changes in coordination geometry and donor identity can be directly monitored. One may then straightforwardly use this method to directly probe anion interactions with the active site metal. Such experiments have been carried out to great advantage for a variety of natural systems such as CA [86] or zinc fingers [87].

In our previous studies of artificial CA, we demonstrated that it is possible to replace the Zn(II) ion in the 3SCC, coordinated by 3 histidine residues and one water or one chloride, with Co(II) and retain ester hydrolysis activity [17]. This substitution strategy provides a powerful means to monitor metal coordination through spectroscopic methods while maintaining catalytic activity, thereby establishing a useful platform for dissecting the rules of anion recognition in designed protein environments. Here we show how small anions interact at neutral pH values with the Co(II) ion bound inside our 3SCC and in one case, explore the pH dependence of such recognition.

Our laboratory has gained experience in the design of metalloproteins for the activation and conversion of small molecules, with a particular emphasis on reactions such as superoxide dismutation [43], nitrite reduction [18,44], and carbon dioxide hydration [45,46]. In these systems, the anions of interest are not only ligands but also reactive substrates that undergo immediate activation once coordinated to the metal. Furthermore, the product of the reaction also may be an anion, such as for the hydrolysis of esters by CA, in which case product inhibition becomes a concern. This intrinsic reactivity, while central to catalytic function, makes it difficult to capture the initial binding interaction. Short lifetimes and fast redox changes at the metal center obscure the distinction between recognition and turnover. As a result, the fundamental parameters that govern anion binding in our systems are often inferred indirectly (for example, through kinetic analysis) rather than observed directly *via* a spectroscopic signature. Using Co(II) allows us for the first time to acquire direct thermodynamic parameters that inform future metalloprotein designs.

Addition of CoSO_4_ to a buffered aqueous solution of (GRW-H)_3_ leads to the formation of Co(II)-(GRW-H)_3_ that exhibits a single weak band at 511 nm (ε ≈ 40 M^−1^ cm^−1^), consistent with a six-coordinate Co(II) site bound by three histidine residues and three water molecules [88]. The addition of nitrite, pseudohalides, and halides show clear changes in the visible spectra of Co(II)-(GRW-H)_3_, in both the energy and molar absorptivity of the Co(II) d-d electronic transitions. In contrast, addition of chloride to an aqueous ${\text{Co}(H_{2}O)}_{6}^{2+}$ solution at neutral pH produces no change in the visible spectra (Fig. S5), confirming that the binding interaction is between the anions and the Co(II) ion bound within the 3SCC scaffold.

Studies of a large number of Co(II) complexes have shown that d-d absorption spectra are so characteristic that the geometry of a complex can be reasonably well predicted from the resultant spectra [88-91]. Moreover, Co(II) complexes displaying pseudo-tetrahedral and distorted five-coordinate geometry absorb at lower energy than six coordinate complexes with weak or moderately strong ligands of similar positions in the spectrochemical series. This is due to the smaller splitting of the d-electron energy levels in pseudo-tetrahedral and distorted five-coordinate geometries. Pseudo-tetrahedral Co(II) complexes, lacking an inversion center, exhibit d-d absorption bands in the visible region with high molar absorption coefficients, generally above 300 M^−1^ cm^−1^, whereas five-coordinate complexes display d-d absorption bands with lower molar absorption coefficients and weak bands of about 5–10 M^−1^ cm^−1^ around 720 nm [50]. In contrast, six-coordinate Co(II) complexes typically show broad d-d bands of even lower intensity (ε lower than 40 M^−1^ cm^−1^) in the visible region, between 400 and 550 nm [88].

The trends for anion binding to the different Co(II)-(GRW-H)_3_ scaffolds can be distinguished using the visible spectra shown in Fig. 2 and reported in Table 1. Among the Co(II)-(GRW-H)_3_ adducts, the chloride complex shows the most distinct tetrahedral character having the largest molar absorption coefficients of the group with ε = 365 M^−1^ cm^−1^ (560 nm), ε = 320 M^−1^ cm^−1^ (588 nm) and ε = 460 M^−1^ cm^−1^ (613 nm). We envision that the moderate size, charge density, and field strength of the chlorine, which complements the preorganized arrangement of histidine residues within (GRW-H)_3_ pocket, likely favors a tetrahedral geometry. The increasing ionic radius and polarizability exhibited by iodide, along with the weaker and more flexible bonds, can no longer accommodate the rigid cavity of the 3SCC in a tetrahedral structure. The conversion from a 4-coordinate to 5 or 6-coordinate center is supported by the increase in number of transitions and the decrease by a factor of 4 to 5 in molar absorption coefficients for these bands. Bromide appears to be an intermediate case, probably with the predominant species being tetrahedral, but with some of the higher coordination environment present. This structural selectivity may be seen as an indirect role of the protein matrix in imposing a geometric constraint on the metal center and on the accessibility of small molecules. While other *de novo* designed metalloproteins have demonstrated that residues surrounding the metal site can influence the coordination geometry [92], the present system provides a clearer, spectroscopically tractable example in which the scaffold itself enforces a defined coordination mode, allowing direct correlation between ligand identity, geometry, and binding affinity.

**Fig. 5.**
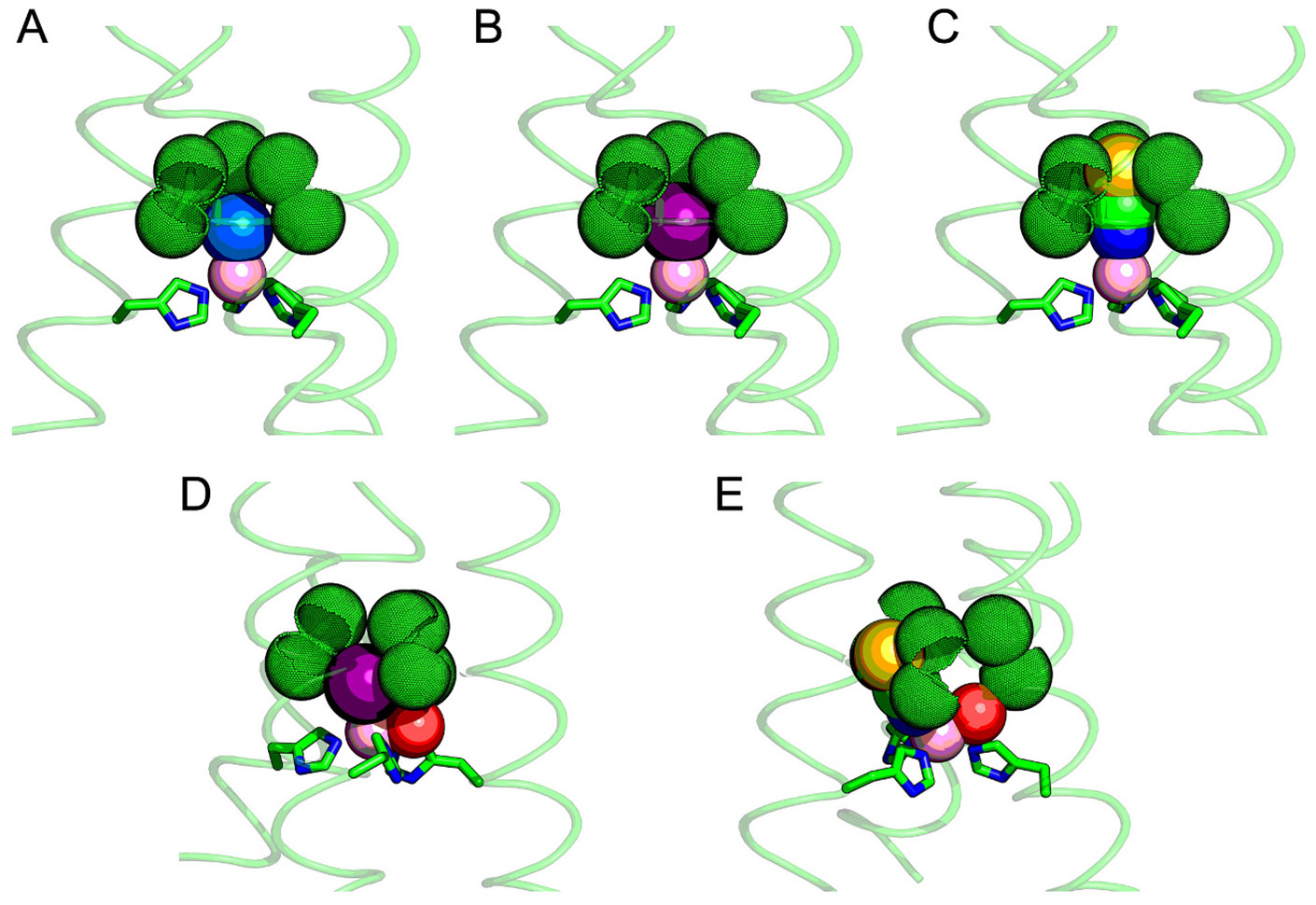
Structural models, derived from the X-ray structure of Pb(II)Zn(II)-GRCS-L16C-L30H (PDB ID: 5kb0), illustrating anion coordination in Co(II)-(GRW-H)_3_. Zoomed views of the Co(II)His_3_ metal site are shown in a tetrahedral geometry with the fourth ligand as (A) chloride, (B) iodide, or (C) thiocyanate (N-bound). Panels (D) and (E) depict pentacoordinate Co(II)His_3_ models, featuring water as the fourth ligand and either (D) iodide or (E) thiocyanate (N-bound) as the fifth ligand. In all panels, Co(II) is shown as a pink sphere, coordinating His30 residues as sticks, anions in sphere representation (blue for chloride; violet for iodide; and, for thiocyanate, blue for nitrogen, green for carbon, and yellow for sulfur), water is shown as a red sphere, and the δ-carbons of Leu26 are indicated as dots to illustrate their spatial orientation around the metal site. The γ-carbons of the Leu26 residues have been removed for clarity.

While the energy and the intensities of the d-d transitions in Co(II)-(GRW-H)_3_-Halide adducts are broadly consistent with those reported for Co(II)-substituted CA, the trend in binding affinities differs markedly. In the natural Co(II)-substituted CA, the affinity follows the order with I^−^ > Br^−^ > Cl^−^ > F^−^ [50], whereas in the 3SCC scaffolds the sequence is inverted (Cl^−^ > Br^−^ ≈ I^−^, with no detectable F^−^ binding). These reversals pinpoint the strong influence of 3SCC hydrophobic interior on metal-anion recognition. In CA, a large and important water channel surrounds the active site, providing both a pathway for substrate and product access and egress, and a hydrophilic environment that stabilizes halide binding. In contrast, the interior of the 3SCC is highly hydrophobic and much more constrained by the leucine layer above the metal binding site that forms a roof at approximately 6.6 Å above the metal. The hydration energy of fluoride is extremely high, and the energetic cost of desolvation outweighs the modest enthalpic stabilization gained upon binding to the hydrophobic Co(II)–3SCC site. As the anion size increases, the hydration penalty decreases (−465 kJ mol^−1^ for F^−^, −340 kJ mol^−1^ for Cl^−^, −315 kJ mol^−1^ for Br^−^, −275 kJ mol^−1^ for I^−^) [93] and binding becomes feasible. The structural models provide insight into how different anions are accommodated within the Co(II)His_3_ metal site of (GRW-H)_3_. Chloride, the smallest halide that binds, fits well in a tetrahedral geometry (Fig. 5A), as its extension from Co(II) (about 3.4 Å^1^) allows it to occupy the fourth coordination position with minimal steric clash against Leu26 residues. In contrast, iodide has both a larger ionic radius and a longer Co─I bond, requiring a spatial extension from Co(II) of roughly 4.5 Å. Therefore, it cannot be efficiently accommodated in the cavity (Fig. 5B) without a significant perturbation to the leucine layer or reorientation of the coordinated histidines. This conclusion is consistent with the preference of iodide for a pentacoordinate Co(II) environment (Fig. 5D), in which the anion shifts toward the helical interface and the fifth position is likely occupied by a water molecule.

Bromide, intermediate in size and hydration energy, adopts a mixture of these 4 and 5 coordinate Co(II) structures. The competition between dehydration energies and structural confinement imposed by the leucine layer required within the helical scaffold yields the observed trends. Thus, the binding becomes tighter for chloride than iodide in the 3SCC system, opposite that seen for *CA*.

While halides show weak and geometry-constrained binding, the pseudohalides interact much more strongly with the Co(II) center. This is true both for native CA and the Co(II)-3SCC, although there is a 100-fold decrease in affinity for the designed system. Nonetheless, the pseudohalide anisotropic charge distribution and stronger donor ability promotes tighter coordination and more pronounced ligand-field perturbations, leading to sharper absorption features.

Addition of azide, a spectroscopic mimic of superoxide, produced a dramatic change in the visible region, with the spectrum evolving into three well-defined transitions at 569, 586, and 620 nm (ε up to 180 M^−1^ cm^−1^). The binding affinity is stronger than halides, with an apparent *K*_D_ of (34 ± 2) mM. Thiocyanate displayed similar behavior, yielding four distinct transitions at 500, 552, 578, and 605 nm (ε up to 280 M^−1^ cm^−1^) and the same apparent *K*_D_ of (33 ± 2) mM. Overall, pseudohalides exhibit significantly higher affinity than halides, which can be attributed to their stronger σ-donor interaction with the Co(II) center. Furthermore, their lower hydration energies [93] reduce the desolvation penalty upon binding, promoting more favorable coordination within the hydrophobic 3SCC environment. Furthermore, the lower molar absorption coefficients are consistent with 5-coordinate active sites. The approximately 400 cm^−1^ shift to higher energies for the NCS^−^ *vs* $N_{3}^{-}$ complexes indicates the thiocyanate provides a stronger field ligand than the azide system, suggesting that the N-bound NCS^−^ linkage isomer is formed as a pentacoordinate structure.

To test this hypothesis, we collected Co K-edge XAS of the Co(II)-3SCC-NCS^−^ complex at pH 9.0. The XANES region displays a pronounced pre-edge feature, consistent with a Co(II) ion in a five-coordinate environment. This increase in pre-edge intensity relative to Co(II)-3SCC (without NCS^−^) supports a decrease in symmetry upon thiocyanate binding. Analysis of the EXAFS region yielded the best fit for a coordination environment consisting of five O/N scatterers at 2.07 Å, consistent with N-bound thiocyanate. Fits including Co─S scattering produced only a modest (~3-fold) improvement in R-factor while introducing twice as many variable parameters and chemically unreasonable Debye–Waller factors and bond distances (*e.g.*, Co─S bond length of 1.75 Å). The coordination number of five inferred from the EXAFS data is consistent with the conclusions drawn from UV–Vis spectroscopy. The structural modeling of the Co(II)-3SCC-NCS^−^ complex described in Fig. 5 further supports this interpretation. Because of its elongated N-C-S structure, the thiocyanate will clash with leucine residues regardless of its geometry; however, attempts to accommodate this anion as fourth ligand in a tetrahedral geometry (Fig. 5C) lead to serious negative contacts with the layer of leucines in the 26 position. In contrast, arranging the NCS^−^ toward the helical interface in a pentacoordinate environment (Fig. 5E) provides more space for thiocyanate to fit within the 3SCC. Still, it is likely that modest rearrangement of nearby Leu26 residues (or the bound histidine residues) is still required.

The final anion tested was nitrite because we have evaluated nitrite reduction for a series of Cu-3SCCs and felt that these binding studies might provide some insight into the selectivity of this coiled coil for this anion. Interestingly, substitution of the native Zn(II) in CA II with Cu(II) has been reported to induce alternative catalytic activities beyond CO_2_ hydration, most notably the reduction of ${\text{NO}}_{2}^{-}$ to NO [94]. Moreover, bovine Zn(II)-CA II has also been proposed to catalyze NO formation from nitrite and to contribute to nitrite-dependent vasodilation, although these findings remain controversial [52,53]. For this reason, evaluating the binding behavior and affinity of nitrite in our Co(II)-3SCC system may provide useful insight into how this anion interacts with metal centers supported by 3-His coordination environments in natural metalloenzymes. Addition of ${\text{NO}}_{2}^{-}$ produced a broad and asymmetric absorption band. Spectral deconvolution resolved two main components at 528 nm (ε = 165 M^−1^ cm^−1^) and 570 nm (ε = 125 M^−1^ cm^−1^). The higher energy of the d-d transitions observed for the nitrite complex compared to those of the NCS^−^ and $N_{3}^{-}$ adducts suggests coordination through nitrogen rather than oxygen. This interpretation is consistent with the stronger ligand field expected for N-bound nitrite and aligns with the spectral features typically associated with Co(II)-N donor interactions. The nitrite ion falls intermediate between the binding affinities of halides and pseudohalides to Co(II). It can be noted that it is thought that the $\text{Cu(I)-}{\text{NO}}_{2}^{-}$ adduct for our designed Cu nitrite reductases is also thought to bind as the N-adduct in this scaffold with *K*_m_ values in the 50–250 mM range [18].

It is important to note that all these spectroscopic and binding studies were performed at pH 9.0, where the non-active esterase catalyst exists, presumably Co(II)(His)_3_(H_2_O)_3−x_(OH^−^)_x_ (with x = 0 or 1). Under more basic conditions, new red-shifted features start to appear in the visible spectra of Co(II)-(GRW-H)_3_ at 590 nm and 640 nm, with slightly higher molar absorption coefficients. We previously assigned this species as a pentacoordinate structure, Co(II)(His)_3_(H_2_O)_2−x_(OH^−^)_x_ (with x = 1 or 2), which exhibits catalytic activity in esterase reactions. Weak or no anion binding is observed when this pentacoordinate hydroxo species is present, perhaps because the coordination environment becomes too rigid and sterically constrained to allow substitution of the remaining aqua ligand.

Because of the differential relative affinities of hydroxide and other anions, we explored the pH-dependent affinity of the strongest-binding anion, thiocyanate. The titration data at different pHs for the reaction:

$${\text{Co(II)(His)}}_{3}{\left(H_{2}O\right)}_{3-x}{\left({\text{OH}}^{-}\right)}_{x}\to {\text{Co(II)(His)}}_{3}(\text{NCS}){\left(H_{2}O\right)}_{2-x}{\left({\text{OH}}^{-}\right)}_{x}.$$

with x = 0 or 1, are shown in Fig. 3. We observed that the affinity for thiocyanate increased modestly going to higher pH from a value of (70 ± 4) mM at pH 7 to (33 ± 2) mM at pH 9. The affinity increase leveled out at this pH and further addition of base led to competition with hydroxide. The apparent pK_a_ for the affinity shift is (8.00 ± 0.09), requiring a single deprotonation event. These data are quite interesting as they appear to provide information regarding the properties of the coiled coil directly. The pK_a_ for NCSH (1.1) is far to acidic to be responsible for this behavior. In contrast, the conversion of Co(II)(His)_3_(H_2_O)_3−x_(OH^−^)_x_ (with x = 0 or 1) to the high pH species Co(II)(His)_3_(H_2_O)_2−x_(OH^−^)_x_ (with x = 1 or 2) is 10.25 and it is known that thiocyanate does not bind to this form. Therefore, this behavior does not appear to be directly related either to the metal or the anion. Given that neither deprotonation of HNCS nor deprotonation at the metal can explain the effect, the modest twofold increase in thiocyanate affinity likely originates from features of the 3SCC scaffold. One possibility is that pH-dependent changes in interhelical interactions, such as partial weakening of Lys-Glu salt bridges at the helical interface, could slightly increase accessibility to the metal site and thereby enhance anion binding. Further work will be required to determine the origin of this pH dependence.

## 5. Conclusions

In this report, we have examined anion binding using our *de novo* designed 3SCC metalloprotein that has been shown previously to be an effective catalyst for small anion transformations. As either the Zn(II) or Co(II) species, this scaffold is capable of ester hydrolysis at pH values above 9 or 10, respectively. At lower pH values both cations are in an inactive form, presumably because the catalytically necessary bound hydroxide ion has been converted to water. While the Zn(II) artificial protein retains the native 4-coordinate geometry of CA, the Co(II) 3SCC adopts a 6-coordinate structure below pH 9. Because Co(II) has a partially filled d shell, we are capable of completing direct spectroscopic monitoring of anions to this enzyme form. Therefore, all binding studies were carried out with the Co(II)-reconstituted protein to interrogate anion interactions with the precursor of the high pH catalytic center. We have not examined the catalytically active high-pH form of the Co(II) center in detail because, at pH 10, hydroxide effectively outcompetes the binding of halides, pseudohalides, or nitrite. None-the-less, the binding of anions to metals in this neutral or mildly basic pH form of this scaffold provides information on how anions are accepted within the substrate binding cavity for related catalytic centers within artificial Cu Nitrite Reductases and superoxide dismutases prepared with the identical GRW-H peptide. At pH values near or below pH 9, nitrite, azide, and thiocyanate bind to Co(II) with millimolar affinities, switching the metal coordination environment from octahedral to pentacoordinate, as assessed by UV–Vis spectroscopy. Moreover, thiocyanate, an ambidentate ligand, forms a five-coordinate Co(II) center through nitrogen-only coordination, as confirmed by XAS. The modest increase in its affinity at higher pH values pinpoints a single deprotonation event likely connected to the protein scaffold rather than to the anion or the metal ion. Halides display distinct behaviors. While no fluoride binding is observed, likely due to its high hydration energy, chloride, bromide, and iodide bind more weakly than pseudohalides and show geometry-dependent trends that are controlled by the protein matrix. Together, these findings demonstrate that a minimal His_3_ metal center engineered in a small and well-defined protein matrix can discriminate between anions through a combination of coordination preferences and protein matrix constraints. In particular, while the GRW-H peptides have a restricted active site/anion binding cavity toward the N terminus of the catalytic center, CA has a much more forgiving water channel that allows all halides to bind similarly to Zn(II) or Co(II). Within a helical structure, the hydrophobic layer located ~ 6.6 Å above the metal site directly influence halide binding in a unique way, changing both metal coordination and halide affinity significantly than what is observed for *CA*. Overall, this work establishes design principles for controlling anion recognition in artificial metalloproteins and pave the way for future efforts to design artificial metalloproteins capable of activating and transforming small inorganic substrates.

## Supplementary Material

1

## Figures and Tables

**Table 1:** Wavelengths (λ) and molar absorption coefficients (ε) obtained from spectral deconvolution of the visible electronic absorption spectra of Co(II)-(GRW-H)_3_ in the presence of different anions, along with the apparent dissociation constants (*K*_D_).

	Co(II)-(GRW-H)_3_		Co(II)-CA [50]	
Anion	λ, nm (ε, M^−1^ cm^−1^)	*K*_D_ (mM)	λ, nm (ε, M^−1^ cm^−1^)	*K*_D_(mM)
${\text{NO}}_{2}^{-}$	528 (165), 570 (125)	(108 ± 2)	–	–
$N_{3}^{-}$	569 (180), 586 (40), 620 (140)	(34 ± 2)	472 (110), 543 (210), 568 (250), 641 (65)	0.25
NCS^−^	500 (40), 552 (168), 578 (55), 605 (280)	(33 ± 2)	465 (100), 529 (90), 571 (100), 690 (9)	0.16
Cl^−^	560 (365), 588 (320), 613 (460), 640 (95)	(545 ± 21)	495 (160), 552 (220), 591 (270), 719 (4)	20
Br^−^	567 (335), 592 (180), 618 (360)	(1108 ± 38)	492 (170), 535 (90), 550 (200), 595 (200), 714 (10),	7.9
I^−^	471 (70), 514 (90), 567 (185), 603 (95), 634 (125)	(1046 ± 36)	498 (190), 529 (180), 552 (170), 600 (120), 629 (80), 757 (9)	1

## Footnotes

1 Estimated from a typical Co─Cl bond length of ~2.2 Å combined with the ionic radius of chloride

## Appendix A. Supplementary data

Supplementary data to this article can be found online at https://doi.org/10.1016/j.jinorgbio.2026.113268.
