# Supplementary material for: Probing anion recognition in a cobalt(II) de novo designed metalloprotein
Source: J Inorg Biochem. Author manuscript; Available in PMC 2026 Jun 12. (PMC13262294; doi:10.1016/j.jinorgbio.2026.113268)
Supplement: 1 [file NIHMS2177819-supplement-1.docx]

**Supporting information**

**Probing anion recognition in a cobalt(II) *de novo* designed metalloprotein**

**Salvatore La Gatta^1^, Jacob K. Firby^1^, James E. Penner-Hahn^1,2^*, Vincent L. Pecoraro^1^***

^1^Department of Chemistry, University of Michigan, Ann Arbor, MI 48109, USA

^2^Department of Biophysics, University of Michigan, Ann Arbor, MI 48109, USA

*Correspondence to: [vlpec@umich.edu](mailto:vlpec@umich.edu) and [jeph@umich.edu](mailto:jeph@umich.edu)

**Table of Contents**

Fig. S1. ESI-MS spectrum and mass deconvolution of GRW-H…………………………………………….2

Fig. S2. Binding isotherms for Co(II)-(GRW-H)_3_ with different anions…………………………………….3

Fig. S3. UV-Vis spectrum of Co(II)-(GRW-H)_3_……………………………………………………………..4

Fig. S4. UV-Vis spectra of Co(II)-(GRW-H)_3_ with increasing fluoride concentrations……………………...4

Fig. S5. UV-Vis spectra of CoCl_2_ with increasing chloride concentrations………………………………….5

Table S1. EXAFS fitting results for Co(II)-(GRW-H)_3_-NCS^–^……………………………………………….6

Table S2. Multishell EXAFS fitting results for Co(II)-(GRW-H)_3_-NCS^–^……………………………………6

**
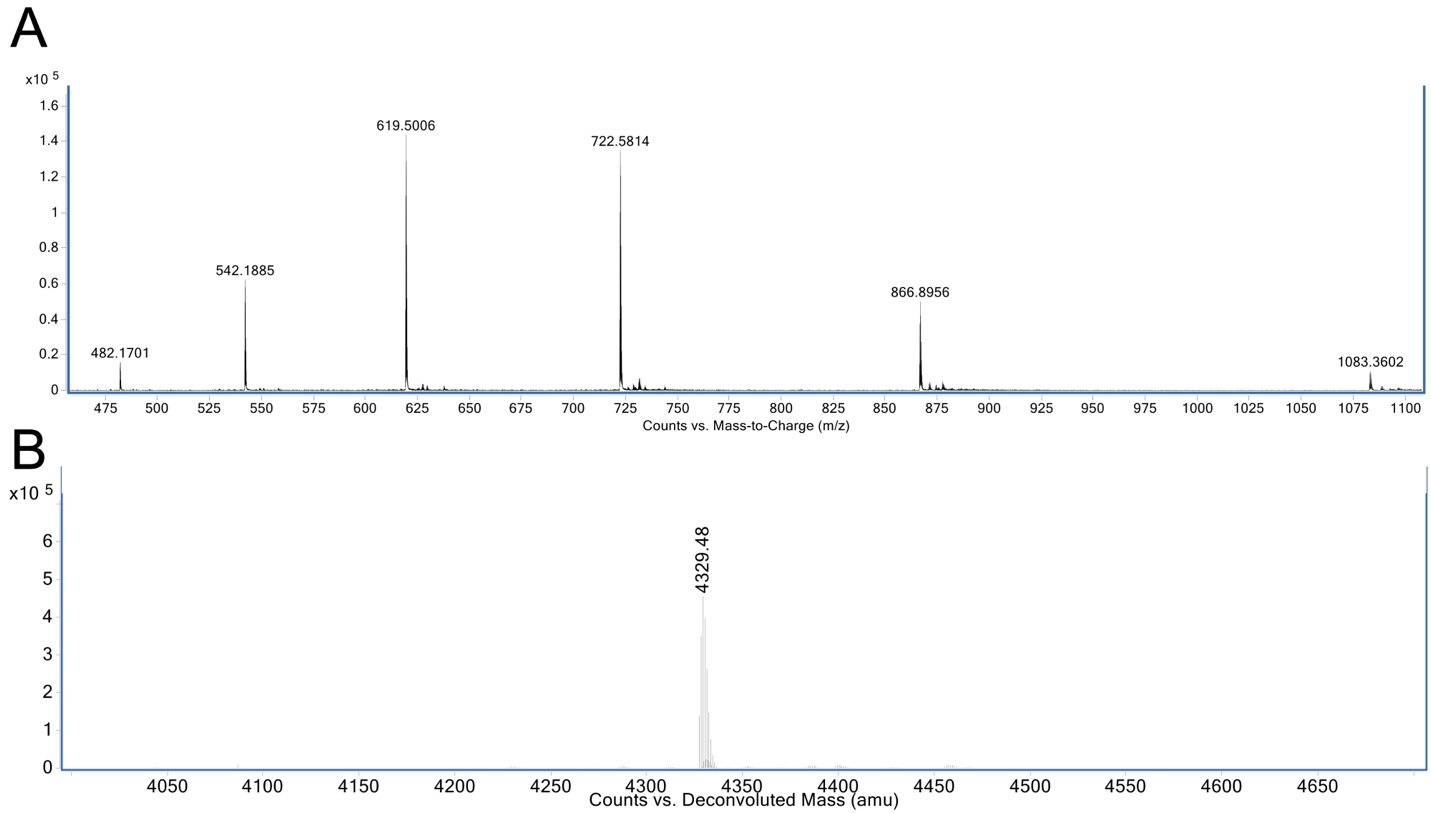
Fig. S1.** (A) ESI-MS spectrum of GRW-H. The signal at m/z = 1083.36 Th corresponds to the [M+4H^+^]^4+^ ion (theoretical average m/z: 1083.37 Th); the signal at m/z = 866.89 Th corresponds to the [M+5H^+^]^5+^ ion (theoretical average m/z: 866.89 Th); the signal at m/z = 722.58 Th corresponds to the [M+6H^+^]^6+^ ion (theoretical average m/z: 722.58 Th); the signal at m/z = 619.50 Th corresponds to the [M+7H^+^]^7+^ ion (theoretical average m/z: 619.50 Th); the signal at m/z = 542.19 Th corresponds to the [M+8H^+^]^8+^ ion (theoretical average m/z: 542.19 Th); the signal at m/z = 482.17 Th corresponds to the [M+9H^+^]^9+^ ion (theoretical average m/z: 482.06 Th). (B) Spectrum deconvolution gave (4329.48 ± 0.01) Da (theoretical mass: 4329.45 Da).

**
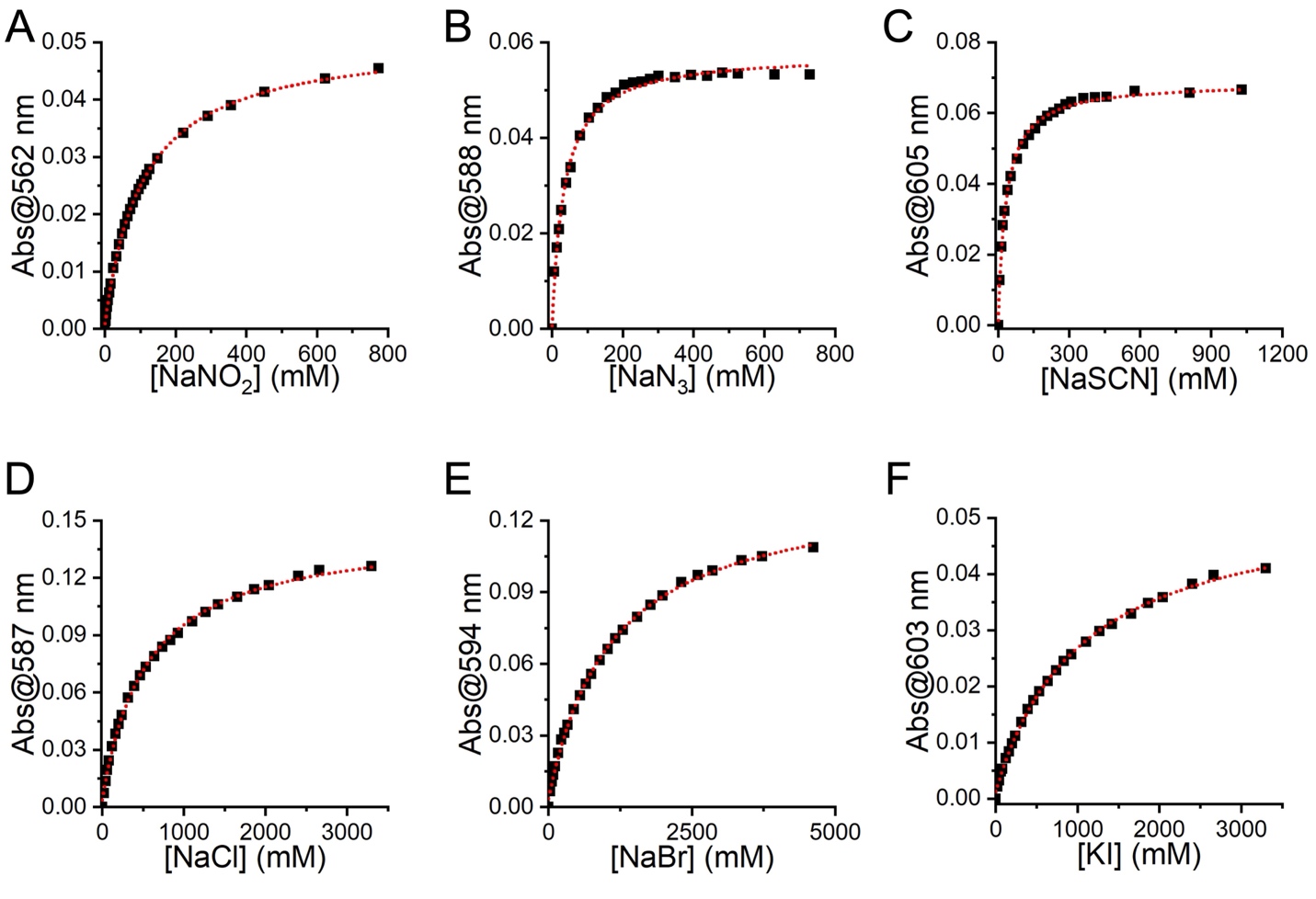
Fig. S2.** Binding isotherms used to determine the apparent dissociation constants (*K*_D_) for Co(II)-(GRW-H)_3_ in the presence of various anions. Each plot shows the change in absorbance as a function of anion concentration, fitted to a single-site binding model to extract the corresponding *K*_D_ values reported in Table 1. Measurements were performed at 0.24 mM Co(II)-(GRW-H)_3_ in 200 mM CHES, pH 9.0.

**
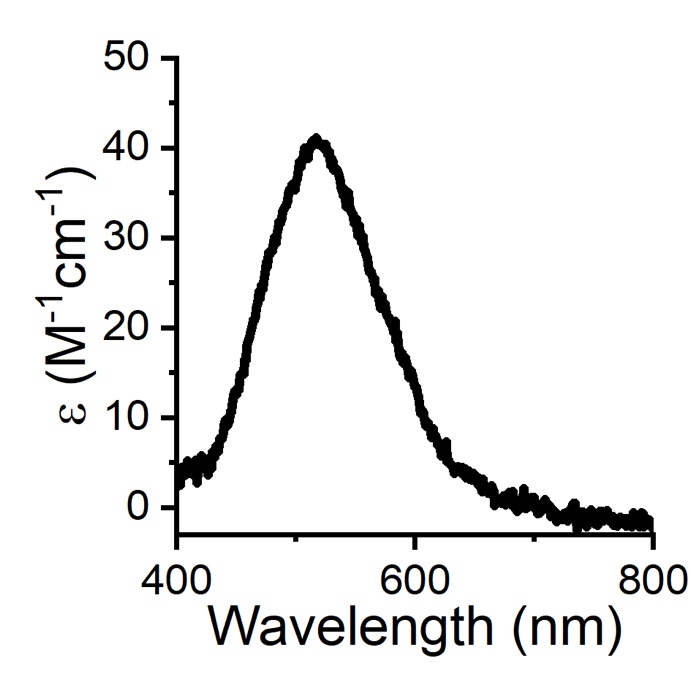
**

**Fig. S3.** UV–Vis spectrum of Co(II)-(GRW-H)_3_ in 50 mM CHES, pH 9.0.


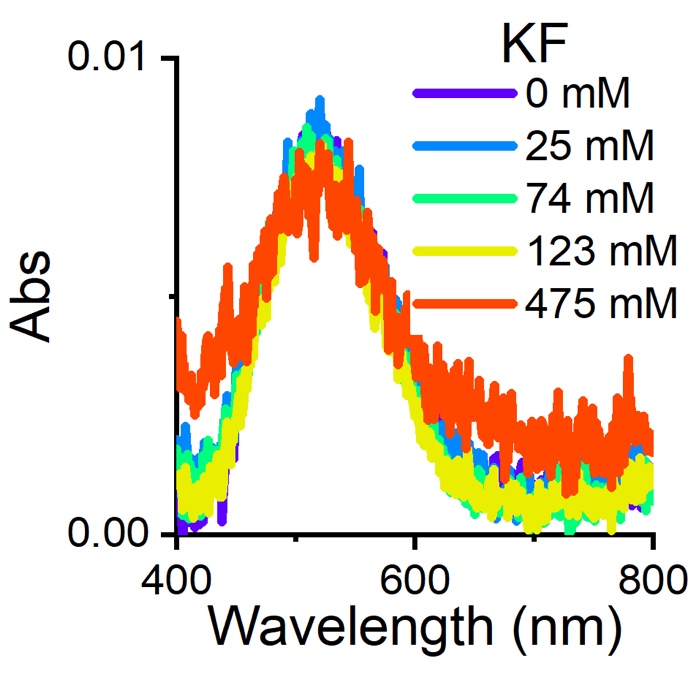


**Fig. S4.** UV–Vis spectra of Co(II)-(GRW-H)_3_ (0.24 mM) in 200 mM CHES, pH 9.0, in the presence of increasing concentrations of KF.


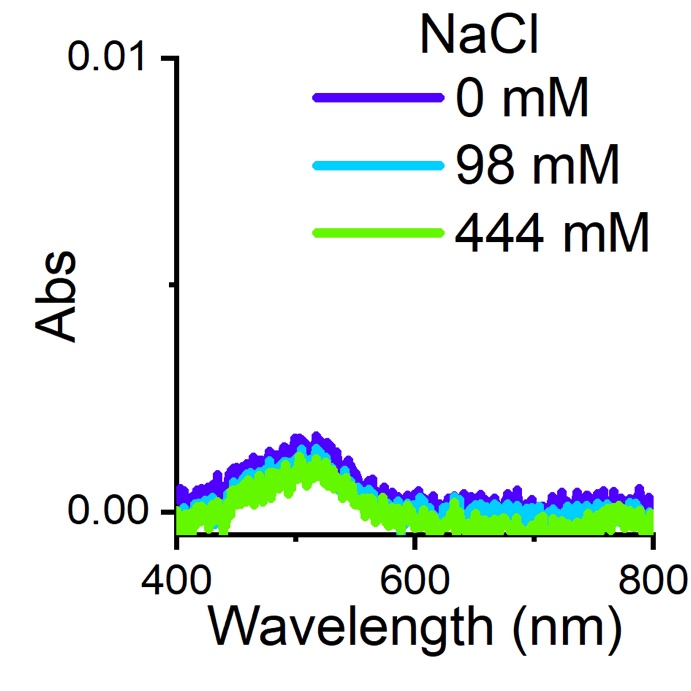


**Fig. S5.** UV–Vis spectra of CoCl_2_ (0.24 mM) in 200 mM HEPES, pH 7.0, upon addition of increasing concentrations of NaCl.

**Table S1.** EXAFS fitting results for Co(II)-(GRW-H)_3_-NCS^–^.

| **Model** | **S0^2^** | **Co-N σ^2^ x 10^–3^ (Å^2^)** | **Co-N/O R (Å)** | **Co-S σ^2^ x 10^–3^ (Å^2^)** | **Co-S R (Å)** | **Reduced chi-square** | **R-factor** |
| --- | --- | --- | --- | --- | --- | --- | --- |
| Co-(N/O)_5_ | 0.51 | 3.5 | 2.073 | – | – | 320 | 0.0967 |
| Co-N_2_S | 0.79 | 0.9 | 2.055 | 17.9 | 1.755 | 154 | 0.0351 |

**Table S2.** Full multishell EXAFS fitting results for Co(II)-(GRW-H)_3_-NCS^–^, corresponding to the first-shell Co-(N/O)_5_ model reported in Table S1 but expanded to a 3 His + 1 O + 1 N-bound NCS^–^ coordination environment and including outer-shell scattering contributions.

| **Path** | **Path degeneracy** | **σ^2^ x 10^–3^ (Å^2^)** | **R (Å)** |
| --- | --- | --- | --- |
| N/O | 5 | 3.5 | 2.073 |
| C (NCS^–^) | 1 | 8.6 | 3.133 |
| N-C (NCS^–^) | 2 | 8.6 | 3.133 |
| N-C-N (NCS^–^) | 1 | 8.6 | 3.133 |
| C (Imidazole) | 6 | 8.7 | 3.041 |
| N-C (Imidazole) | 12 | 8.7 | 3.221 |
| N-C-N (Imidazole) | 6 | 8.7 | 3.400 |
| N-N (Imidazole) | 6 | 8.7 | 4.208 |
| N-C (Imidazole) | 6 | 8.7 | 4.218 |
| C-N (Imidazole) | 6 | 8.7 | 4.285 |
